# Oxidative stress generated by polycyclic aromatic hydrocarbons from ambient particulate matter enhance vascular smooth muscle cell migration through MMP upregulation and actin reorganization

**DOI:** 10.1186/s12989-022-00472-z

**Published:** 2022-04-22

**Authors:** Sujin Ju, Leejin Lim, Young-Jae Ki, Dong-Hyun Choi, Heesang Song

**Affiliations:** 1 Department of Biochemistry and Molecular Biology, Chosun University School of Medicine, Gwangju, 61452 Korea; 2 Cancer Mutation Research Center, Chosun University, Gwangju, 61452 Korea; 3 Department of Internal Medicine, Chosun University School of Medicine, Gwangju, 61452 Korea

**Keywords:** Ambient particulate matter, Polycyclic aromatic hydrocarbons, Oxidative stress, Vascular smooth muscle cells, Migration, Matrix metalloproteinase

## Abstract

**Background:**

Epidemiological studies have suggested that elevated concentrations of particulate matter (PM) are strongly associated with the incidence of atherosclerosis, however, the underlying cellular and molecular mechanisms of atherosclerosis by PM exposure and the components that are mainly responsible for this adverse effect remain to be established. In this investigation, we evaluated the effects of ambient PM on vascular smooth muscle cell (VSMC) behavior. Furthermore, the effects of polycyclic aromatic hydrocarbons (PAHs), major components of PM, on VSMC migration and the underlying mechanisms were examined.

**Results:**

VSMC migration was significantly increased by treatment with organic matters extracted from ambient PM. The total amount of PAHs contained in WPM was higher than that in SPM, leading to higher ROS generation and VSMC migration. The increased migration was successfully inhibited by treatment with the anti-oxidant, N-acetyl-cysteine (NAC). The levels of matrix metalloproteinase (MMP) 2 and 9 were significantly increased in ambient PM-treated VSMCs, with MMP9 levels being significantly higher in WPM-treated VSMCs than in those treated with SPM. As expected, migration was significantly increased in all tested PAHs (anthracene, ANT; benz(a)anthracene, BaA) and their oxygenated derivatives (9,10-Anthraquinone, AQ; 7,12-benz(a)anthraquinone, BAQ, respectively). The phosphorylated levels of focal adhesion kinase (FAK) and formation of the focal adhesion complex were significantly increased in ambient PM or PAH-treated VSMCs, and these effects were blocked by administration of NAC or α-NF, an inhibitor of AhR, the receptor that allows PAH uptake. Subsequently, the levels of phosphorylated Src and NRF, the downstream targets of FAK, were altered with a pattern similar to that of p-FAK.

**Conclusions:**

PAHs, including oxy-PAHs, in ambient PM may have dual effects that lead to an increase in VSMC migration. One is the generation of oxidative stress followed by MMP upregulation, and the other is actin reorganization that results from the activation of the focal adhesion complex.

## Background

Epidemiological studies have shown a positive correlation between exposure to air pollution and the risk of carotid atherosclerosis. For example, in people who have had long-term exposure to indoor coal-burning pollution, an elevation of inflammatory cytokines, such as IL-8, CRP, and TNF-α, has been observed, leading to a significant increase in the risk of carotid atherosclerosis [1]. In addition, the concentration of PM2.5 in ambient pollution is positively correlated with carotid intima-media thickness and the development of high-risk coronary plaques [2, 3]. Indeed, the correlation between air pollution and the risk of atherosclerosis has been documented in most of the epidemiological studies conducted to date [2, 4, 5]. Several studies have also shown that exposure to PM or diesel exhaust particles (DEP) alters the expression levels of atherosclerosis-related proteins, the levels of MAPK phosphorylation, or caused the development of plaques in arterial walls in animal models [6, 7].

Ambient PM, composed of natural and anthropogenic particles, is an airborne mixture of substances including organic and inorganic compounds such as metals and biological components [8]. There is growing evidence that polycyclic aromatic hydrocarbons (PAHs) and their derivatives, such as oxygenated- (oxy-PAHs), nitro-, hydroxyl-, and chloro-PAHs, comprise a major part of air pollution that is correlated with the increase in cardiovascular morbidity and mortality [9–11]. In particular, PAHs and oxy-PAHs, which are generated by many combustion processes in urban environments, are major components of air pollution [12, 13]. In addition, oxy-PAHs also originate from reactions between PAHs and various forms of organic and inorganic radicals [14], or from the photo-oxidation of PAHs by singlet molecular oxygen [15]. It is well known that PAHs and oxy-PAHs induce severe redox stress in cells and tissues, leading to the oxidation of nucleic acids, proteins, and lipids [16, 17] as well as carcinogenicity [18, 19]. In a previous study, we showed that in cardiomyocytes, oxy-PAHs induced higher electrophysiological instability than PAHs, which might be due to the increased generation of ROS. In addition, we showed that the amount and number of oxy-PAHs contained in WPM were higher in SPM, leading to higher electrophysiological instability [20].

Vascular smooth muscle cells (VSMCs), constituting the major cells in the media layer of arteries, participate in arterial wall remodeling, which plays an important role in atherosclerosis throughout all stages of the disease. VSMCs proliferation and migration from the media into the intima is believed to be one of the main causes of the formation of atherosclerotic plaques and intimal hyperplasia [21]. However, few studies have investigated the underlying mechanisms in the vasculature, which is composed of endothelial cells (ECs) and vascular smooth muscle cells (VSMCs). For example, in ECs, cellular glutathione is protective against the cytotoxic effects induced by benzo[a]pyrene-1,6-quinone, a PAH component of air pollution [22]. In addition, benzo[a]pyrene increase VSMC migration and invasion through the upregulation of matrix metalloproteinases (MMPs) [23]. Nevertheless, further cellular and molecular alterations in VSMCs induced by ambient PMs and the components that participate in these alterations, and their underlying mechanisms still need to be elucidated.

## Results

### Organic matters extracted from ambient PM induces VSMCs migration through ROS generation

**Fig. 1:**
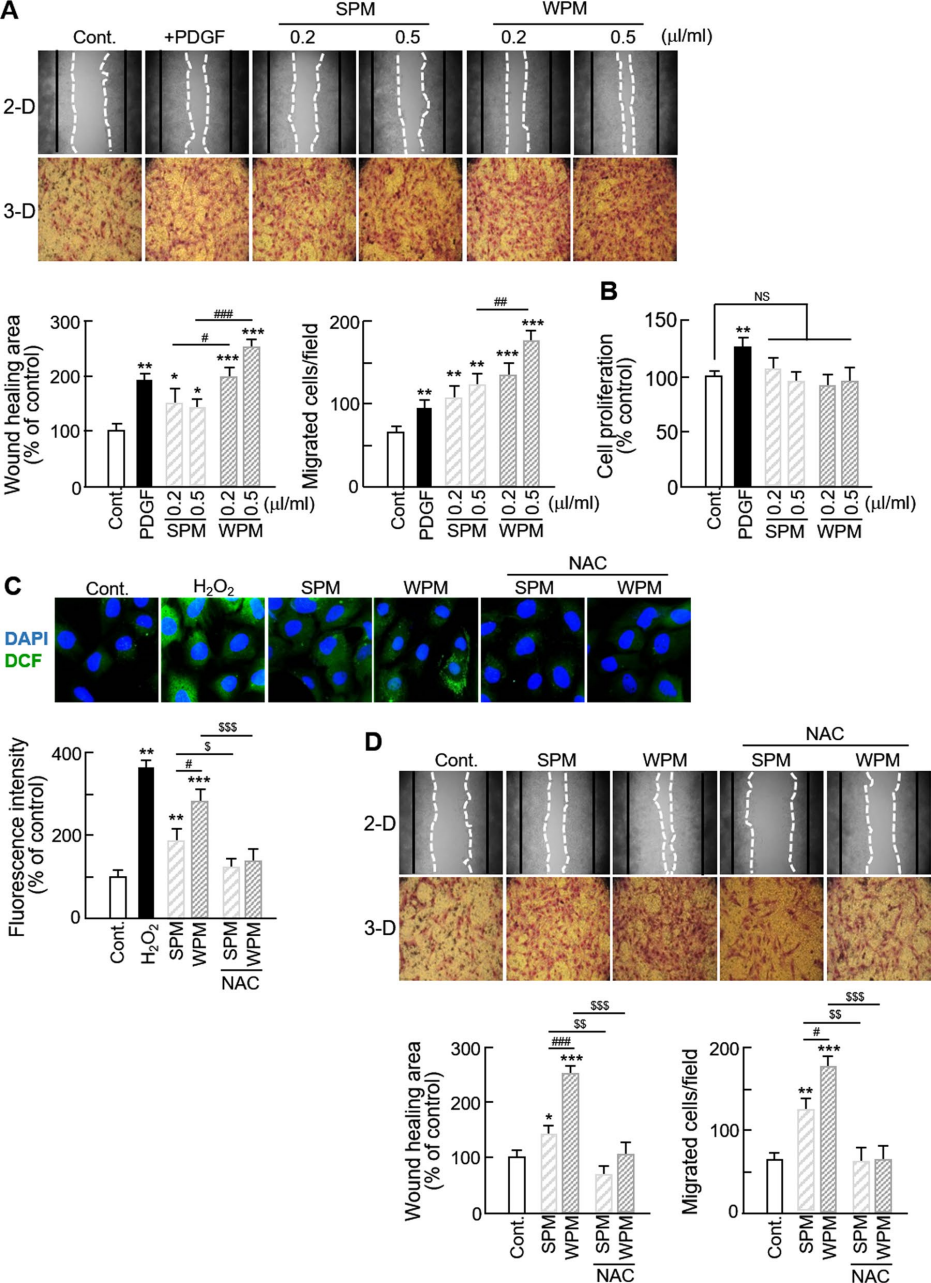
Effect of organic matters from extracted ambient PM on VSMC migration. **a** Effects of seasonal organic matters from ambient PM on VSMC migration were measured using a wound healing assay (upper) and Boyden chamber assay (lower). The images from the wound healing assay are representative of five independent experiments, taken at the time of scratching and 48 h after scratching. The black line represents the initial boundaries following the scratch at time 0 and the white dotted line represents the migrating cell front after 48 h. The wound healing area was measured using ImageJ software. Images from the Boyden chamber assay shows the number of migrated cells. The images from the transwell assay are representative of five independent experiments, taken 12 h after seeding. **b** The effects of ambient PM on VSMC proliferation were evaluated using the BrdU incorporation assay. Serum-starved VSMCs were treated with negative control (0.1% DMSO), SPM, WPM or PDGF-BB (20 ng/mL) as a positive control for 24 h. **c** ROS generation in PM-treated VSMCs was evaluated using H_2_DCF-DA (green). VSMCs were pretreated with or without NAC (1 mM) for 1 h, then treated with negative control (0.1% DMSO), SPM or WPM. Nuclei were stained with DAPI (blue). Fluorescence intensity was quantified by SIBIA software. **d** Effects of the antioxidant, NAC, on organic matters from ambient PM-induced VSMC migration were measured using a wound healing assay (upper) and Boyden chamber assay (lower). All values are represented as mean ± SD. *P < 0.05, **P < 0.01, and ***P < 0.001 versus control; ^#^P < 0.05, ^##^P < 0.01, and ^###^P < 0.001 WPM versus SPM; ^$^P < 0.05, ^$$^P < 0.01, and ^$$$^P < 0.001 PM with NAC versus PM alone; NS, no significance

To investigate the effects of organic matters extracted from ambient PM on VSMC behavior, we analyzed the alterations in VSMC proliferation and migration. As shown in Fig. 1a, both PM collected in summer (SPM) and winter (WPM) significantly increased VSMC migration in both the 2-D wound healing assay and the 3-D Boyden chamber assay. The increased migration was significantly higher in WPM-treated VSMCs than in those treated with SPM. In addition, we observed that the increased level of migration was even higher than that of PDGF-treated VSMCs (a positive control). However, VSMC proliferation was not altered by treatment with organic matters from PM (Fig. 1b), demonstrating that organic matters from PM only changes the migratory activities of VMSCs. Based on previous reports that demonstrate that ROS may mediate VSMC migration [24, 25], we evaluated ROS generation and their effects on VSMC migration. We observed that ROS generation was significantly increased in organic matters from PM-treated VSMCs, with a larger increase observed in cells treated with WPM than those treated by SPM. Furthermore, NAC treatment significantly reduced ambient PM-induced ROS levels (Fig. 1c) and subsequently inhibited VSMC migration (Fig. 1d). These results demonstrate that organic matters from PM induce VSMC migration through ROS generation.

### Organic matters extracted from ambient PM regulates matrix metalloproteinase expression and activity

**Fig. 2:**
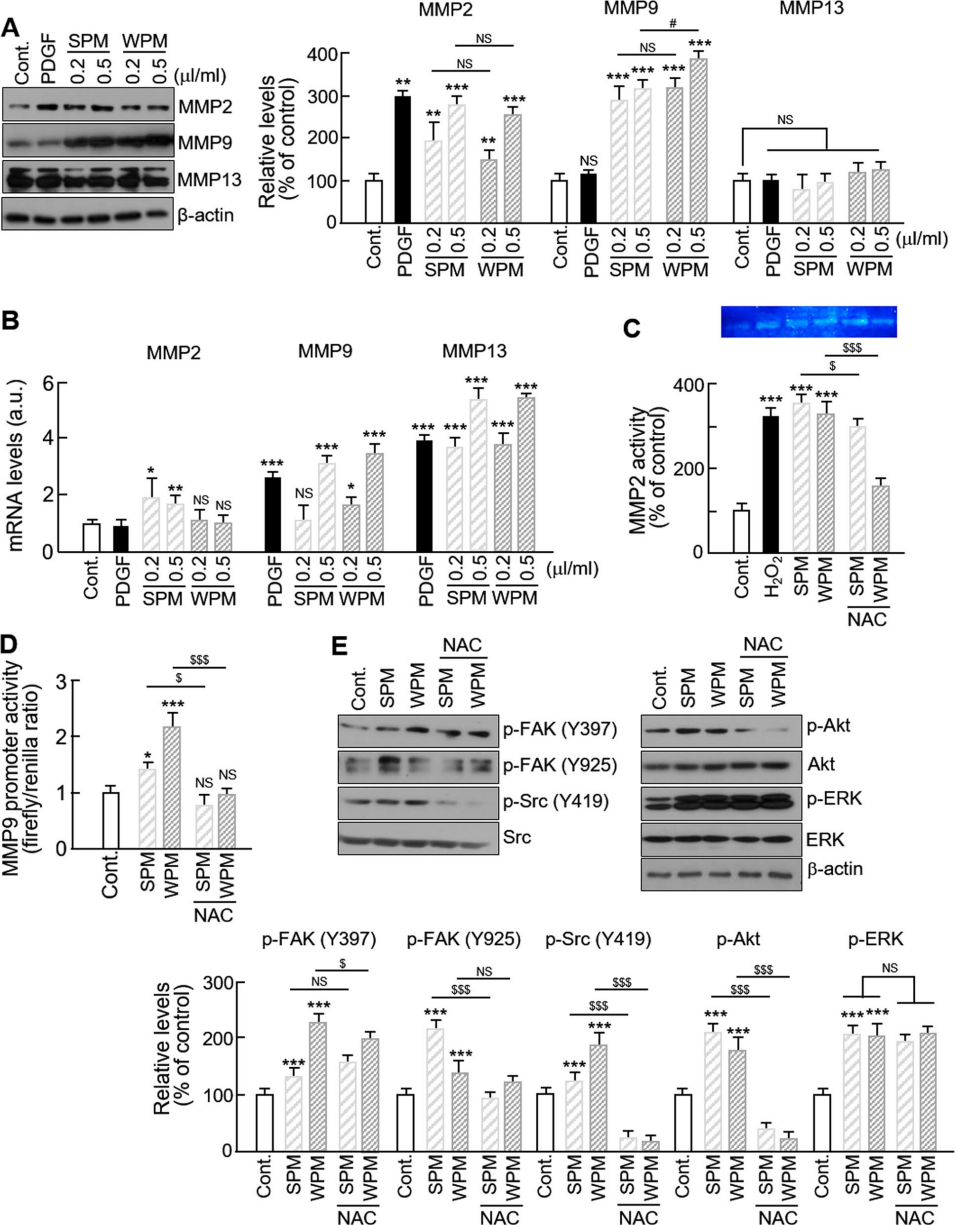
Effect of organic matters extracted from ambient PM on MMP expression and activity in VSMCs. **a** The expression levels of MMP2, 9, and 13 were analyzed by western blotting. Serum-starved VSMCs were treated with control (0.1% DMSO), PDGF-BB (20 ng/mL), SPM, or WPM at the indicated concentration for 24 h. The band densities were normalized to β-actin band density. The gel images shown are representative of those obtained from at least three independent experiments. **b** The mRNA levels of MMPs were quantified by qPCR. Gene expression was normalized to GAPDH. **c** The zymolytic activity of MMP2 was evaluated using gelatin zymography. Serum-starved VSMCs without or with NAC (1 mM) were pretreated with the designated concentrations of SPM or WPM for 24 h. PDGF treatment alone was used as a positive control. The media were then collected and used for this assay. The images shown are representative of those obtained from at least three independent experiments. **d** The effect of NAC on transcriptional activity of the MMP9 promoter in VSMCs was evaluated using a luciferase assay. **e** The expression levels of phosphorylated ERK1/2, Akt, FAK, and Src were analyzed by western blotting. Protein levels were normalized to their total levels. All values are represented as mean ± SD. *P < 0.05, **P < 0.01, and ***P < 0.001 versus control; ^#^P < 0.05 WPM versus SPM; ^$^P < 0.05 and ^$$$^P < 0.001 PM with NAC versus PM alone; NS, no significance

The altered levels of MMPs in organic matters from PM-treated VSMCs were investigated because MMPs are critical regulators of VSMC migration [26]. As shown in Fig. 2a, VSMCs treated either with SPM or WPM significantly increased their expression of MMP2 and MMP9 proteins in a dose-dependent manner, with a greater increase observed in WPM-treated VSMCs than those treated with SPM, which was consistent with the results obtained from the migratory activity experiment. However, the protein levels of MMP13, one of the effectors of cell migration, were not changed, which demonstrates that organic matters from PM induced VSMC migration through MMP2 and MMP9. As expected, in VSMCs treated with organic matters from PM, we observed a significant increase in the mRNA levels of MMP2 and MMP9, but also in the mRNA levels of MMP13, which is inconsistent with the lack of change in its protein levels (Fig. 2b). In addition, the extracellular proteolytic activity of MMP2 and the transcriptional levels of MMP9 were significantly increased in organic matters from PM-treated VSMCs, and these changes were blocked by NAC treatment (Fig. 2c, d). We also investigated the effects of organic matters from PM on the phosphorylation levels of focal adhesion kinase (FAK) and FAK-related signals since FAK acts as a regulator of MMP expression [27, 28] and also enhances cell migratory activity itself through actin reorganization [29]. The phosphorylation levels of FAK at the Y397 and Y925 sites, and of Src, a downstream target of FAK, were increased by organic matters from PM; however, NAC blocked phosphorylation of p-Src and p-FAK at Y925, but not at Y397. In addition, the phosphorylated levels of Akt and ERK were significantly increased in organic matters from PM-treated VSMCs; yet, NAC inhibited the increase of p-Akt without affecting the levels of p-ERK (Fig. 2e). These results demonstrate that organic matters from PM enhances not only MMP expression, but also FAK phosphorylation, leading to VSMC migration.

### PAHs are mediators inducing ROS generation and VSMCs migration

**Fig. 3:**
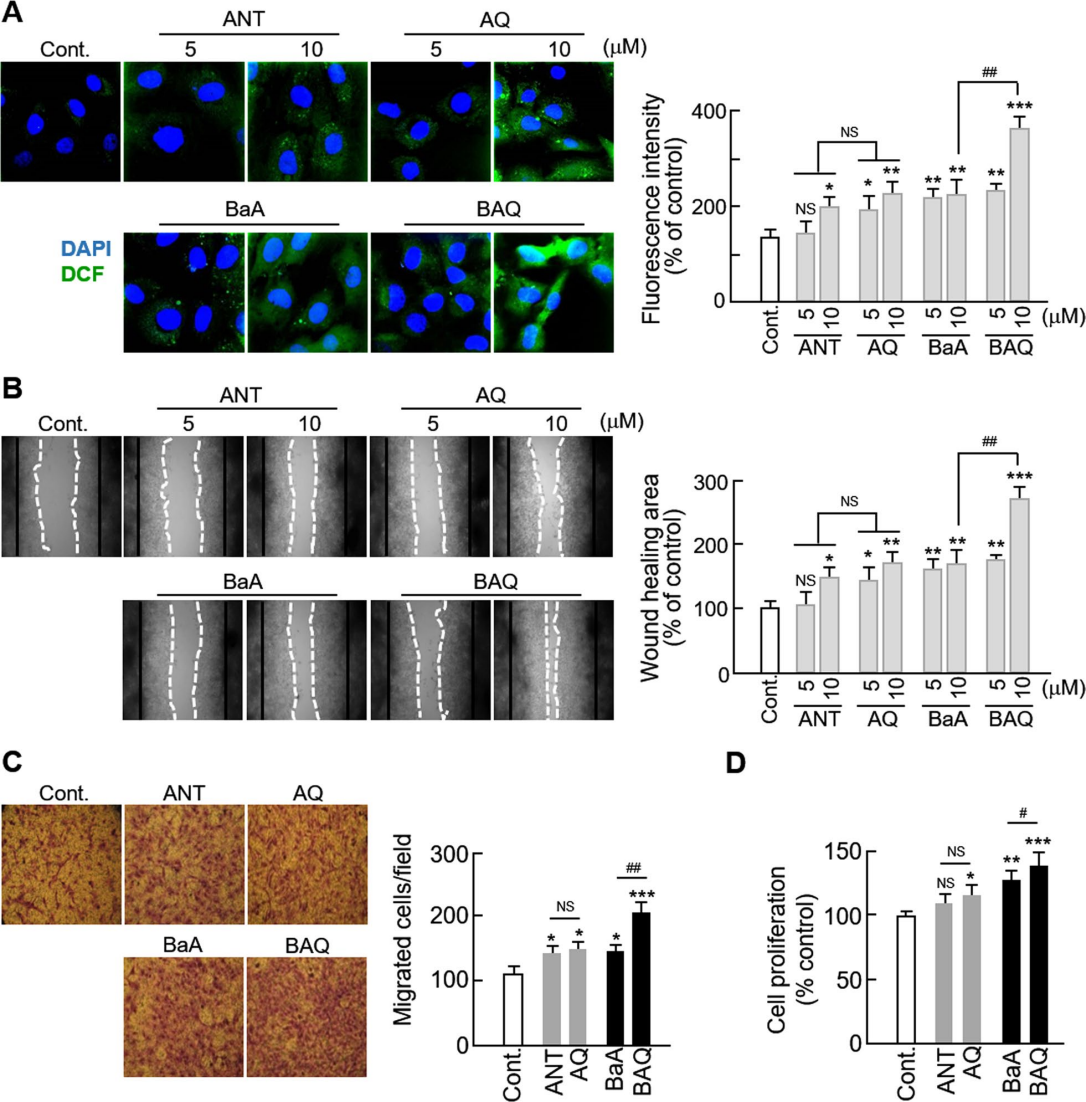
Effect of PAHs on ROS generation and VSMCs migration. **a** VSMCs were treated with control (DMSO), ANT, AQ, BaA, or BAQ at the indicated concentration and analyzed for ROS generation. Representative fluorescent images of VSMCs loaded with H_2_DCF-DA (green) with nuclei stained with DAPI (blue) are shown. **b** Effects of ambient PM on VSMC migration were measured using a wound healing assay. The images from the wound healing assay are representative of five independent experiments, taken at the time of scratching and 48 h after scratching. The black line represents the initial boundaries following the scratch at time 0 and the white dotted line represents the migrating cell front after 48 h. The wound healing area was measured using ImageJ software. **c** Boyden chamber assay showing the number of migrated cells. The images from the transwell assay are representative of five independent experiments, taken 12 h after seeding. **d** The cytotoxic effects of PAHs on VSMCs were evaluated using the MTT assay. Serum-starved VSMCs were treated with each form of PAHs for 24 h. All values are represented as mean ± SD. *P < 0.05, **P < 0.01, and ***P < 0.001 versus control; ^#^P < 0.05 and ^##^P < 0.01 oxy-PAHs versus PAHs; NS, no significance

We have previously suggested that PAHs and oxy-PAHs that are found in ambient PM might act as ROS generators [20]. Therefore, in this study, we hypothesized that PAHs, including oxy-PAHs, comprise the main components of ambient PM that induce ROS generation, which subsequently promotes the migration of VSMCs. We investigated the effects of two types of PAHs, anthracene (ANT) and benz(a)anthracene (BaA), and their oxygenated derivatives, 9,10-anthraquinone (AQ) and 7,12-benz(a)anthraquinone (BAQ), respectively, on ROS generation and VSMC migration. We first demonstrated that PAHs or oxy-PAHs used at concentrations of 5 and 10 µM had no significant cytotoxicity in VSMC (data not shown). As shown in Fig. 3a, VSMCs treated with PAH or oxy-PAH significantly increased their ROS generation in a dose-dependent manner, compared to that of the untreated control. As expected, we observed that the alterations were significantly greater in VSMCs treated with oxy-PAHs than in those treated with PAHs. In addition, VSMC migration was increased by PAH and oxy-PAH treatments in both wound healing and Boyden chamber assays; however, the only significant difference observed between PAHs and oxy-PAHs was between VSMCs treated with BaA and those treated with BAQ, and only at a concentration of 10 μM (Fig. 3b, c). Interestingly, cell proliferation was significantly increased in AQ-, BaA-, and BAQ-treated VSMCs, but not in ANT-treated cells (Fig. 3d). Thus, we have shown that PAHs enhanced VSMC migration.

### PAHs regulates matrix metalloproteinase expression and activity through ROS generation

**Fig. 4:**
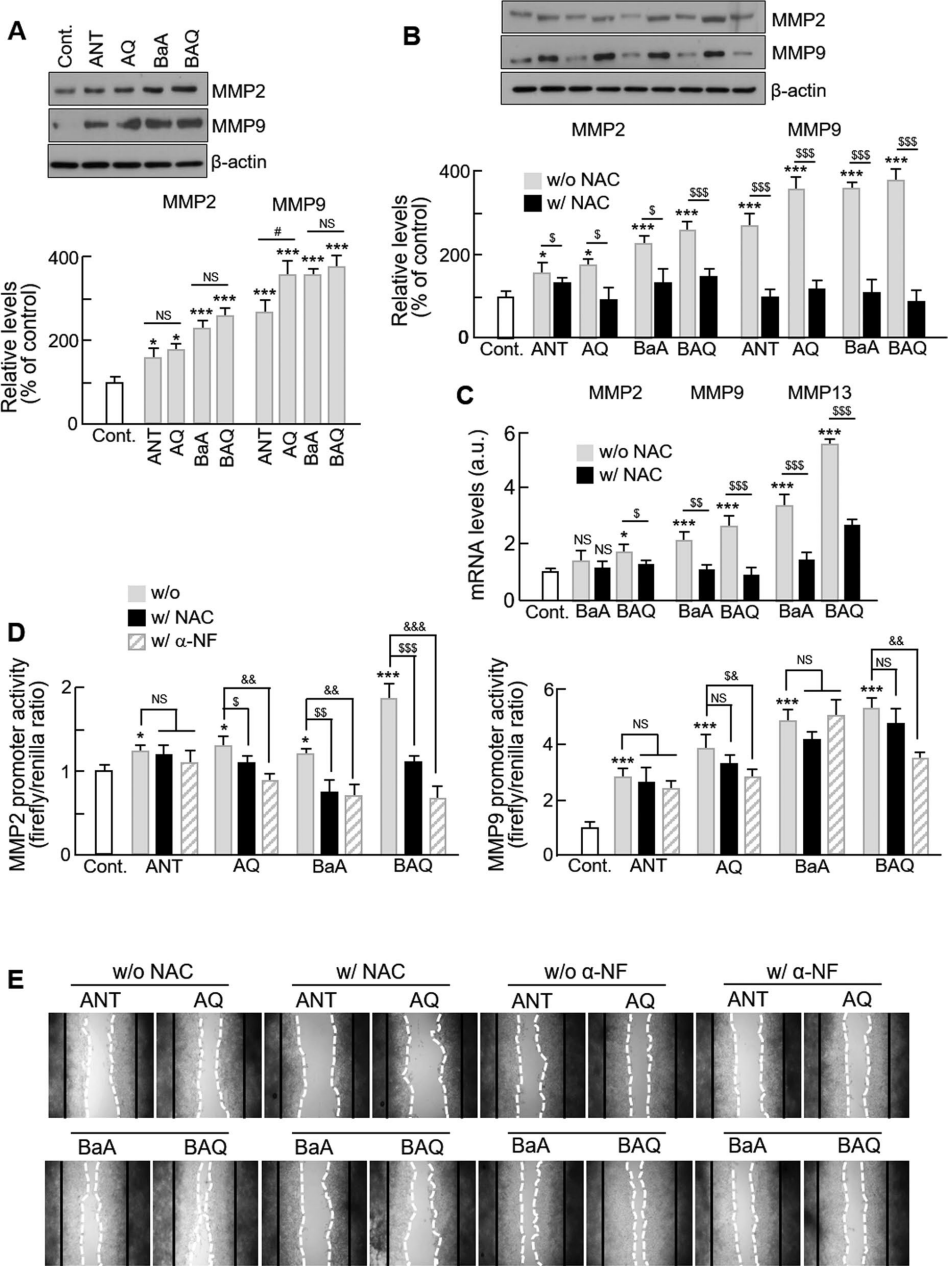
Effect of PAH and oxy-PAH on MMPs expression and VSMCs migration. **a** The expression levels of MMP2 and MMP9 were analyzed by western blotting. Serum-starved VSMCs were treated with control (0.1% DMSO), PAHs or oxy-PAHs for 24 h. The band densities were normalized to β-actin band density. The gel images shown are representative of those obtained from at least three independent experiments. **b** The effect of NAC treatment on the expression levels of MMPs was quantified by western blot. Same volume of water was added in control group for NAC treatment. **c** The mRNA levels of MMPs in VSMCs following the treatment of NAC were quantified by qPCR. Gene expression was normalized to GAPDH. **d** The transcriptional activity of the MMPs promoter was evaluated using a luciferase assay in VSMCs with or without NAC or α-NF treatment. Same volume of DMSO was added in control group for α-NF treatment. Control values of NAC or α-NF were converted to 1 for comparing with the experimental values. **e** The effects of NAC or α-NF on VSMCs migration were evaluated using a wound healing assay. The wound healing area was measured using ImageJ software. The images from the wound healing assay are representative of five independent experiments, taken at the time of scratching and 48 h after scratching. The black line represents the initial boundaries following the scratch at time 0, and the white dotted line represents the migrating cell front after 48 h. All values are represented as mean ± SD. *P < 0.05 and ***P < 0.001 versus control; ^#^P < 0.05 oxy-PAH versus PAH; ^$^P < 0.05, ^$$^P < 0.01, and ^$$$^P < 0.001 PAHs with NAC versus PAHs alone; ^&^P < 0.05, ^&&^P < 0.01, and ^&&&^P < 0.001 PAHs with α-NF versus PAHs alone; NS, no significance

To investigate whether the increased migration by PAHs is due to the altered MMP expression induced by ROS, we evaluated the effects of PAHs on MMP expression. We observed that following treatment with all four types of PAHs, protein expression of MMP2 and MMP9 were significantly increased compared to that of the control (Fig. 4a), with the resulting levels of MMP9 protein being significantly higher than that of MMP2. The only significant difference in MMP9 protein expression between PAHs and oxy-PAHs was when comparing VSMCs treated with ANT to those treated with AQ. The increase in MMP2 and MMP9 were successfully inhibited by NAC treatment (Fig. 4b). Compared to the control, the mRNA levels of MMP2, MMP9, and MMP13 were all significantly increased in BAQ-treated VSMCs, while only MMP9 and MMP13 mRNA were increased in BaA-treated cells. All observed increases in mRNA expression were inhibited by NAC treatment (Fig. 4c). As shown in Fig. 4d, the transcriptional activities of MMP2 and MMP9 were significantly increased in VSMCs treated with all four types of PAHs, and consistent with the results of protein expression, the resulting levels of MMP9 were higher than those of MMP2. In addition, these increases were significantly inhibited by NAC treatment. Moreover, we observed that the increase of transcriptional activity of these MMPs by PAHs was also significantly inhibited by α-naphthoflavone (α-NF), a specific inhibitor of AhR (Fig. 4d). The aryl hydrocarbon receptor (AhR) is a ligand-activated transcription factor that regulates biological responses to planar aromatic hydrocarbons and acts primarily as a sensor of xenobiotic chemicals [30, 31]. Consistent with these findings, the VSMC migration induced by PAHs was also successfully blocked by NAC or α-NF treatment (Fig. 4e). These results demonstrate that PAHs enhance VSMC migration through ROS generation.

### PAHs regulate the expression of ROS-related genes and the dependent signaling pathways

**Fig. 5:**
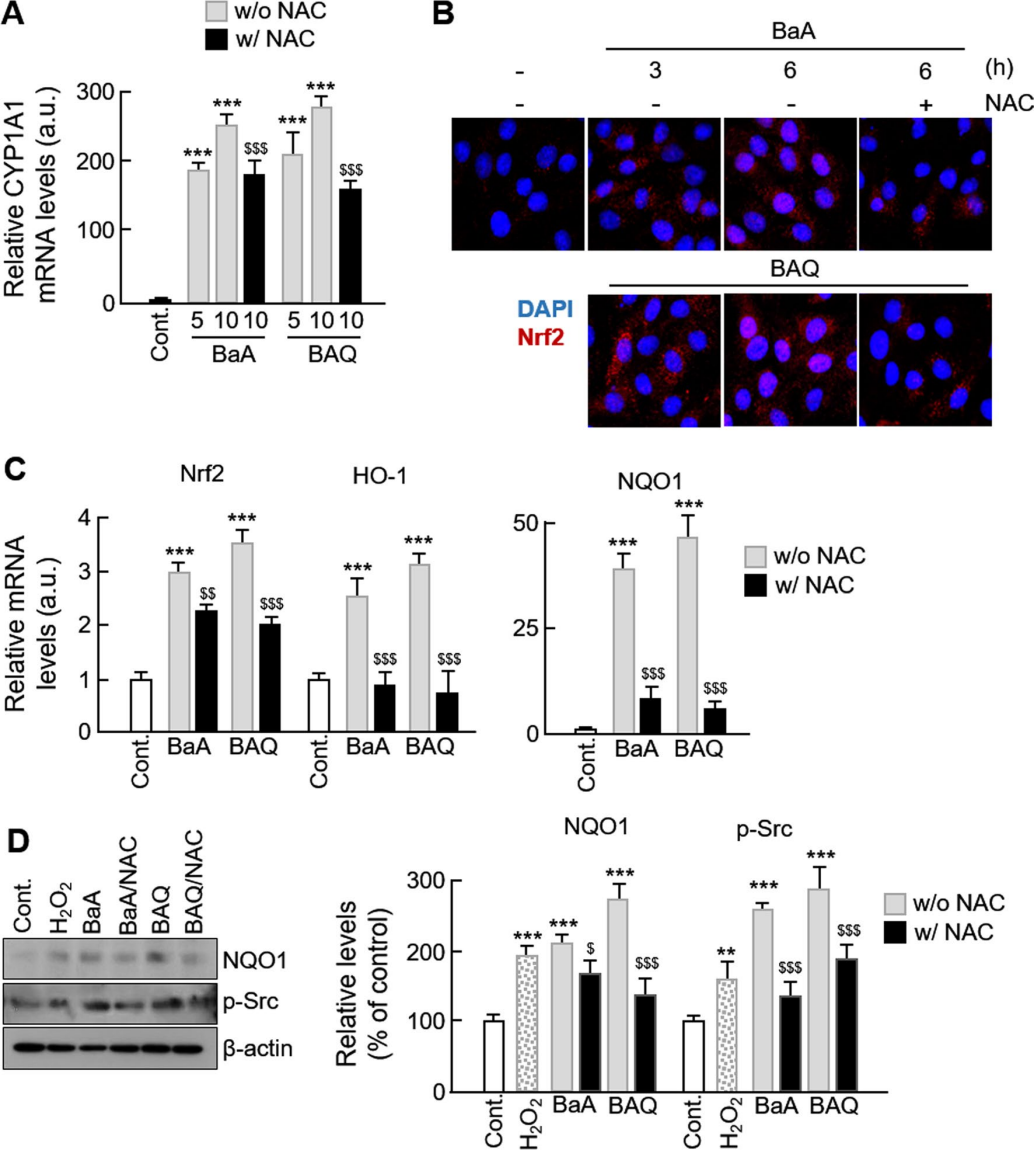
Effect of PAHs and oxy-PAHs on the expression of ROS-dependent signals. **a** The mRNA levels of CYP1A1 in the absence or presence of NAC were evaluated by qPCR. **b** The effect of NAC treatment on the nuclear translocation of Nrf2 was evaluated by immunocytochemistry. **c** The mRNA levels of Nrf2, HO-1, and NQO1 in the absence or presence of NAC were evaluated by qPCR. **d** The expression levels of NQO1 and phosphorylated Src were analyzed by western blotting. Protein levels were normalized to β-actin. All values are represented as mean ± SD. ***P < 0.001 versus control; ^$^P < 0.05, ^$$^P < 0.01, and ^$$$^P < 0.001 PAHs with NAC versus PAHs alone

Since there is evidence that CYP1A1 and transcription factor Nrf2 are protective against PAHs and ambient PM-induced pro-oxidative damage in various cell types [32, 33], we investigated their alterations in PAH-treated VSMCs. The mRNA levels of CYP1A1 were dramatically increased in BaA- and BAQ-treated VSMCs in a time-dependent manner, and these alterations were only partially, but significantly, inhibited by NAC treatment (Fig. 5a). In addition, PAH treatment caused translocation of Nrf2 into the nucleus, and this effect was inhibited by NAC (Fig. 5b). The mRNA levels of Nrf2 and its target genes, heme oxygenase-1 (HO-1) and NAD(P)H dehydrogenase 1 (NQO1), were significantly increased following treatment with both forms of PAHs (Fig. 5c). The protein expression level of NQO1 was also increased by PAH treatment (Fig. 5d). Studies have shown that HO-1 and NQO1 are expressed in an ROS-dependent manner [34], and consistent with the results of these studies, NAC inhibited the increases in mRNA and protein levels. In addition, the phosphorylation level of Src, a downstream target of Nrf2, was significantly increased by PAHs (Fig. 5d). These results demonstrate that the cellular uptake of PAHs generates ROS and regulates the ROS-dependent signaling pathways in VSMCs.

### PAHs enhance the formation of focal adhesion complex through aryl hydrocarbon receptor

**Fig. 6:**
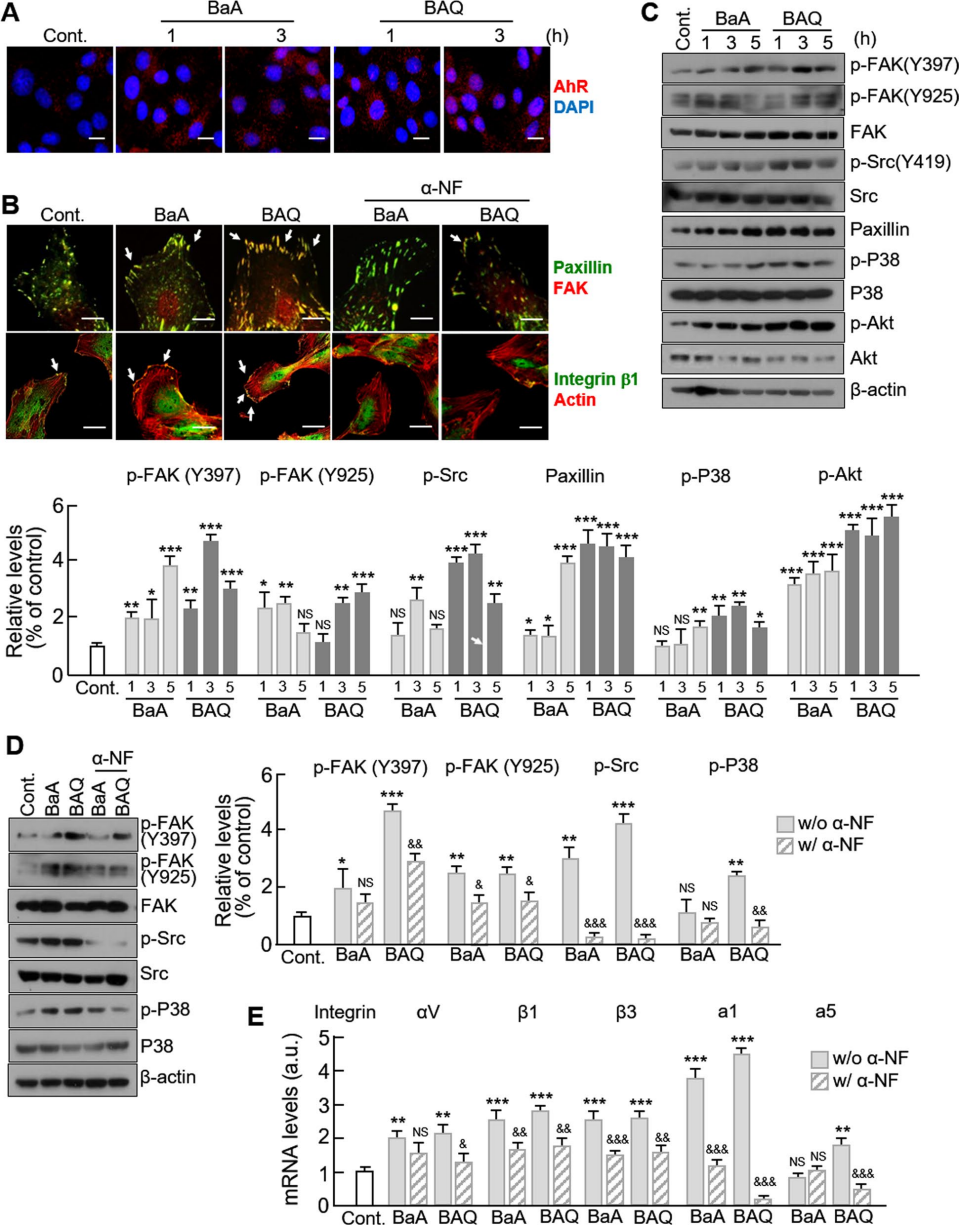
Effects of PAHs and oxy-PAHs on the formation of the focal adhesion complex and actin reorganization. **a** VSMCs were treated with control (0.1% DMSO), 10 μM of ANT, AQ, BaA, or BAQ, and stained with the anti-AhR antibodies (red) at the indicated time points. Representative fluorescent images show the translocation of AhR in the nucleus. Scale bar, 50 µm. **b** The formation of the focal adhesion complex by PAHs was determined by immunocytochemical co-staining for FAK and paxillin; integrin β1 and actin. Representative fluorescent images of VSMCs with and without α-NF treatment are shown. Scale bar, 50 µm. **c** The expression levels of focal adhesion-related proteins were analyzed by western blotting at indicated time points. Protein levels were normalized to their total levels with the exception of paxillin, which was normalized to β-actin. **d** The effect of α-NF on the expression of focal adhesion-related proteins were analyzed by western blotting. Protein levels were normalized against those of β-actin. **e** The effect of α-NF treatment on the mRNA levels of integrins were evaluated by qPCR. All values are represented as mean ± SD. *P < 0.05, **P < 0.01, and ***P < 0.001 versus control; ^&^P < 0.05, ^&&^P < 0.01, and ^&&&^P < 0.001 PAHs with α-NF versus PAHs alone; NS, no significance

The aryl hydrocarbon receptor (AhR) is the primary sensor of aromatic hydrocarbons and is involved with the regulation of focal adhesion (FA) sites [35]. AhR activation subsequently activates Src and FAK, leading to the reorganization of the actin cytoskeleton, which is an effector for the changes observed in cell migration [28]. We first observed that in VSMCs treated with either the PAH BaA or the oxy-PAH BAQ, AhR translocated into the nucleus from the membrane in a time-dependent manner. AhR translocation was significantly higher in BAQ-treated VSMCs than in BaA-treated VSMCs when comparing the observations at the same time points (Fig. 6a). Further co-immunostaining showed that compared to that of controls, the degree of colocalization of FAK and paxillin at the sites of filopodia growth were higher in both forms of PAH-treated VSMCs. Furthermore, BAQ-treated VSMCs displayed more extensive colocalization with paxillin than in BaA-treated cells (Fig. 6b). In addition, we could observe the more spots of integrin β1 on edge of actin cytoskeleton. Blocking of AhR by α-NF significantly reduced the level of these colocalization, suggesting that PAHs affect FA formation and cytoskeletal organization in VSMCs through this receptor. In addition, we confirmed that the levels of phosphorylated FAK and FA-related proteins, such as p-Src and paxillin, were significantly increased in VSMCs treated by both forms of PAH (Fig. 6c). As expected, PAH treatment of VSMCs significantly increased the levels of p-Akt and significantly altered the levels of phosphorylated p38 (Fig. 6c). These alterations were successfully blocked by treatment with α-NF (Fig. 6d). Furthermore, we examined the mRNA levels of integrins αV, β1, β3, a1, and a5, which are the components of FA complexes in the filopodia. With the exception of a5 in VSMCs treated with BaA, all integrin mRNA levels were significantly increased compared to controls in PAH-treated cells. All of these increases were blocked by treatment with α-NF, except for αV in VSMCs treated with BaA (Fig. 6e). Our results indicate that PAH uptake by AhR regulates FA-related biological responses in VSMCs.

## Discussion

Despite the culmination of evidence suggesting that there is an association between ambient air particles and vascular dysfunction, the underlying mechanisms are complex and variable, and remain to be elucidated. Moreover, due to the complexity of PM components, the specific components responsible for PM-induced vascular dysfunction should be investigated.

We have previously reported that ambient PM generates a substantial amount of ROS, which may induce electrophysiological instability in cardiomyocytes [20]. In addition, we revealed that this effect might be caused by PAHs, the main component of PM. The present study demonstrated that PM exposure significantly increased ROS generation in VSMCs, leading to enhanced migratory activity. The significant increase in migration was greater in VSMCs treated with WPM than in those treated with SPM, and the higher ROS generation by WPM was evidenced by the higher levels of oxy-PAHs contained in WPM than in SPM, consistent with the results of our previous report. ROS generation by BAQ, one of the oxy-PAHs tested in this study, was significantly higher than that of its non-oxygenated form, BaA. Furthermore, we observed that VSMC migration was positively correlated with ROS levels. However, unlike BaA and BAQ pair, any significant differences in ROS generation and cell migration between ANT and AQ were observed, which might be due to their different chemical natures and experimental concentrations.

Although VSMC proliferation is also one of the key factors triggering vascular pathology, we did not observe any significant alterations in VSMC proliferation by ambient PM. However, the significant increase in VSMC proliferation following PAHs treatment (except for ANT) tested in this study suggests that other components in ambient PM might participate in regulating the pathways that lead to VSMC proliferation. Another plausible explanation for this discrepancy is that the concentration of PAHs tested in this study was slightly higher than the concentration of mobilized PAHs from PM.

Cell migration requires the fine spatial–temporal integration of many proteins that regulate the fundamental processes responsible for driving cell movement [36]. It includes the various MMPs that degrade extracellular matrix components and cytoskeleton-related proteins that enhance cell protrusion. Furthermore, there is a substantial amount of evidence to suggest that redox stress is associated with cardiovascular pathologies, such as neointima hyperplasia during restenosis [37], angiotensin II-induced hypertension [38], and impaired endothelium-dependent vasorelaxation [39]. Although the mitochondrial respiratory chain is the major source of intracellular ROS in animal cells, VSMCs contain various sources of ROS, including xanthine oxidase, lipoxygenase, nitric oxide synthases, and NADPH oxidases [40]. In addition, ROS can impact the signaling of a variety of molecular targets that participate in VSMC migration, including target proteins associated with extracellular matrix degradation [41], the formation of FA complexes [42], and cytoskeleton dynamics [43].

The findings of this study show that ROS generation by organic matters from PM or PAHs is positively correlated with MMP expression. In addition, the phosphorylated levels of FAK, as well as the levels of FA complex-related proteins, such as paxillin and p-Src, were significantly increased. The successful inhibition of these alterations by the anti-oxidant, NAC, demonstrates that organic matters from PM mediates VSMC migration through ROS generation and that PAHs contained in ambient PM are responsible for these responses. Moreover, our results showed that the mRNA and protein expression levels of MMP2 and MMP9 were significantly increased in SPM, WPM or PAH-treated VSMCs, which is consistent with previous reports that ROS can directly or indirectly activate MMP2 and MMP9 in VSMCs [41, 44]. However, although we demonstrated that MMP13 participates in VSMC migration in our previous report [45], and mitochondrial ROS generation mediates MMP13 expression in unmyelinated axons [46], we did not observe significant alterations in protein levels of MMP13 in SPM, WPM- or PAH-treated VSMCs in this study. This discrepancy might be due to insufficient ROS generation or different cell specificities. Therefore, in future studies, it will be interesting to further examine the effects of ROS on MMP13 expression and VSMC migration. Moreover, the increased transcriptional activity of MMPs and the activated FA complex-related proteins by PAHs was also significantly inhibited by α-naphthoflavone (α-NF), an inhibitor of AhR. Alpha-NF is not only an antagonist of the Ah receptor (AhR), but is also used as an inhibitor of aromatase and cytochrome P450. Although α-NF has multiple targets, both AhR and cytochrome P450 (CYP1A1, CYP1A2/1B1) inhibition by α-NF are well known to inhibit the metabolic activation of several environmental xenobiotics such as PAHs. Moreover, CYP1A1 is classically known to be regulated by the AhR. In addition, α-NF is used as an antagonist of AhR to confirm the association with the transcriptional activity of AhR by xenobiotics [47]. Consistent with these previous results, the treatment of α-NF significantly inhibited PAHs uptake into cells, leading to a decrease in the transcriptional activities of MMPs and VSMCs migration.

Our results also demonstrated that organic matters from PM or PAHs increased the phosphorylation of FAK and FA-related proteins. Indeed, FA dynamics (assembly and disassembly) involving the coordination between the FA and the actin cytoskeleton is a required process for cell migration [48]. Furthermore, integrins mediate the dynamic interactions between the extracellular matrix and the actin cytoskeleton during cell movement [49]. During this process, the interaction between integrins α5β1 and αVβ3, and FAK acts as a key mediator for VSMC migration [50]. Moreover, there is convincing evidence that FA and actin cytoskeleton dynamics are directly regulated by ROS [51, 52]. In agreement with the above reports, our results showed that the binding between actin and integrin β1 in the FA complex was increased in the filapodia region in PAH-treated VSMCs. In addition, the formation of FA complex was also increased in PAH-treated VSMCs.

Furthermore, we observed that PAH treatment led to significant activation and nuclear translocation of the transcription factor Nrf2 and subsequently increased the expression of the target proteins of Nrf2, HO-1, and NQO1. The mRNA levels of CYP1A1 were also significantly increased in PAH-treated VSMCs. Our results concur with a previous report that showed that exposure to diesel exhaust particulate matter led to significant activation of Nrf2 and expression of CYP1A1 in endothelial cells [32]. However, the correlation between ROS levels and these alterations was not evaluated in this previous study. Although Nrf2 activation and the upregulation of CYP1A1 are protective against ROS insult, the significant increase in VSMC migration in our study suggests that the level of ROS generation following PM or PAH treatment may be beyond their protective capacity.

The present study has some methodological limitations. First, one of the drawbacks is that PAHs are not the only components responsible for ROS generation in PM. Cationic metals, including Fe, Zn, Mn, and Cu, contained in ambient PM are known to produce ROS in biological systems. In particular, there is convincing evidence to suggest that Fe generates ROS through the Haber–Weiss and Fenton reaction [53]. Indeed, we observed that ambient PM contained these metallic compounds in our previous study [20]. Therefore, further investigation of the association between metals and PAHs that lead to ROS-mediated VSMC migration is needed. Second, as this was an *in* vitro study, the consequences of organic matters from PM or PAH treatment reported here may not manifest in humans following real-world inhalation. However, the detected amount of total PAHs including oxy-PAHs in organic matters from PM was 4.1 or 26.12 ng/m^3^ in summer PM (SPM) or winter PM (WPM), respectively in our previous study. Therefore, the equivalent amount to cubic meters of air to 5 µM PAHs, ANT, AQ, BaA, and BQ were 0.22, 0.24, 0.27, and 0.31 m^3^/ml, respectively, to SPM and 0.03, 0.04, 0.04, and 0.05 m^3^/ml to WPM, which demonstrated that the amount of PAHs in real air is enough to induce hazardous effects. Finally, because vascular pathology is mediated by complicated interactions between endothelial cells and VSMCs, the effects of PM or PAHs on endothelial cells need to be evaluated. Despite the limitations of this study, our results on the effects of ambient PM or PAHs on VSMC migration may still be relevant when examining mammalian vascular pathophysiology.

## Conclusions

Our results provide strong evidence of the underlying mechanism of action for PM-induced progression of atherosclerosis. We demonstrated that organic matters from PM increased VSMC migration by ROS generation, and PAHs are the major contributors to this effect. We further showed that ROS generated by PM or PAHs affects VSMC migration in two ways. First is through the significant increase of MMPs, which causes the degradation of extracellular matrix, and second is the formation of the FA complex that allows for actin cytoskeleton rearrangement. Although our findings of vascular pathology caused by ambient PM is supported by an increasing amount of clinical evidence, the in vivo and clinical relevance of these findings remain to be elucidated.

## Methods

### Ambient particulate matters and preparation of organic components

Collection of ambient particulate matter (PM) at Seoul metropolitan area during summer (SPM) and winter (WPM) season and particle preparation was described in the previous study [54], which showed the detailed process for the sampling of PM10 and extraction of organic matter from the PM. For further usage, the extracts were diluted tenfold in dimethylsulfoxide (DMSO). The analyzed organic compounds including PAHs and oxy-PAHs and their concentrations were presented in the previous study [20].

### Isolation of VSMCs and treatments

All animal experiments for the isolation of rat aortic VSMCs were conducted in accordance with the International Guide for the Care and Use of Laboratory Animals. The protocol was approved by the Animal Research Committee of the Chosun University School of Medicine (Protocol No. CIACUC2020-S0032). Rat aortic VSMCs were isolated from 6-week-old Sprague–Dawley rats as described previously [55]. Briefly, the removed aorta was freed from connective tissues and blood clots, then severed and transferred into a tube containing a mixture of collagenase type I (1 mg/mL, Sigma, St. Louis, MO, US) and elastase (0.5 mg/mL, Worthington, NJ, US) and incubated for 30 min at 37 °C. The aorta was placed into a 100-mm cell culture dish and the adventitia was stripped with forceps under a binocular microscope. Each piece of the aorta was transferred into a tube containing 5 mL of enzyme dissociation mixture (containing collagenase and elastase), and the tubes were incubated for 2 h at 37 °C. The dispersion of the tissue was accomplished by slow pipetting. The suspension was centrifuged (1600×*g* for 5 min), and the obtained pellet was resuspended in DMEM with 10% fetal bovine serum (FBS, WelGENE, KOREA). This dissociation step was repeated until tissues were completely dispersed. The cells were cultured at 37 °C in an incubator with a humidified atmosphere of 95% air and 5% CO_2_ and used for up to 10 passages in this study. The cells were then treated with the designated volumes of organic matters from PM and 0.1% of DMSO at a final concentration is used as control. VSMCs were pretreated with or without 1 mM of NAC or 10 nM of α-NF for 1 h before exposure to organic matters from PM, PAHs, or oxy-PAHs. NAC or α-NF were dissolved in PBS or DMSO, respectively.

### Measurement of cytotoxicity and cell proliferation

Cytotoxicity was determined by MTT assay using a CellTiter 96 Assay kit (Promega) according to the manufacturer’s instructions. Briefly, VSMCs were seeded into a 96-well plate in triplicate and cultured for 12 h, and then cells were treated with PAHs or oxy-PAHs for 24 h. The absorbance was measured at 490 nm using an ELISA reader (TECAN, infinite M200 PRO). The proliferative rates of VSMCs were evaluated by determining BrdU incorporation using the BrdU Cell Proliferation Assay kit (Cell signaling). VSMCs were treated with seasonal ambient PM, PDGF-BB (20 ng/ml), PAHs, or oxy-PAHs for 24 h. Then cells were incubated with BrdU for 4 h and subsequent procedure was performed according to the manufacturer’s instructions. The absorbance was measured at 450 nm using an ELISA reader (TECAN, infinite M200 PRO).

### Cell migration assay

Cell migration was examined by three-dimensional Boyden chamber assay and two-dimensional wound healing assay. For Boyden chamber assays, cells (5 × 10^4^ cells in 100 μl) were placed in the upper compartment of the transwell chambers coated with collagen I on the lower surface. Negative control (DMSO), positive control (PDGF, 20 ng/ml), organic matters from PM, PAHs or oxy-PAHs were treated with the designated concentration in each upper compartment with designated concentration. After incubation for 16 h at 37 °C, cells on the lower surface of the filter were fixed and stained, and five random fields/membranes were counted at × 200 magnifications. For wound healing assay, a rectangular lesion was created using a cell scraper, and then the cells were incubated with a designated concentration of organic matters from PM, PAHs or oxy-PAHs for designated times. The distance from the margin of the lesion to the most migrated cells was measured, and the mean value of the distances was taken as the mobility of cells in each culture dish.

### Measurement of intracellular reactive oxygen species (ROS)

Intracellular ROS were measured using the fluorescent dye technique. VSMCs were seeded into a 24well plate with glass coverslips at a density of 5 × 10^4^ cells/ml and cultured for 24 h. Then, cells were treated with negative control (0.1% DMSO), positive control (200 nM of H_2_O_2_), ambient PM, or PAHs in a dose dependent manner for 1 h. Then, cells were washed twice with calcium-free PBS (PBSc) and loaded with 2′,7′-dichlorofluorescein diacetate (H_2_DCF-DA, Invitrogen, USA) and 4′,6-diamidino-2-phenylindole (DAPI) diluted with calcium-free warm PBS to a final concentration of 10 μM and 50 μg/ml, respectively. After, cells were incubated for 10 min at 37℃ in dark. The probe H_2_DCF-DA (10 μM) entered into the cells, and the acetate groups on the H_2_DCF-DA were cleaved by cellular esterases, trapping the nonfluorescent 2′,7′-dichlorofluorescein (DCFH) within the cells. Subsequent oxidation by reactive oxygen species yielded the fluorescent product DCF. Then, cells were gently washed the coverslips three times in warm PBS and the coverslips were placed in the chamber, which was mounted on the stage of an inverted microscope equipped with a confocal laser-scanning system. The dye, when exposed to an excitation wavelength of 480 nm, emitted light at 535 nm only when it had been oxidized. Fluorescence images were collected using a confocal microscope (Fluoview FV1000 confocal system, Olympus) by excitation at 488 nm and emission greater than 500 nm with a long-pass barrier filter. The fluorescence intensity of an equivalent field size (3 × 3 mm) in the plate was measured using the Image J quantification software.

### Zymography

Briefly, aliquots of the control and test media were electrophoresed on a 10% SDS–polyacrylamide gel containing 0.8% gelatin or 0.3 mg/ml collagen. Gels were washed with 2.5% Triton X-100 to remove SDS for 1 h, washed with D.W for 1 h and then incubated at 37 °C for 48 h in developing buffer (gelatin incubation buffer: 50 mM Tris–HCL, pH 7.5, 5 mM CaCl_2_, 1 µM ZnCl_2_, 0.02% sodium azide, 1% Triton X-100 or collagen incubation buffer: 50 mM Tris–HCL, pH 7.5, 10 mM CaCl_2_, 50 mM NaCl, 0.05% Brij35, pH 7.6). After 48 h, the gel was stained with 1% Coomassie blue for 1 h and later, then destained until there is a good resolution between the bands and blue background. The gel was photographed and zymolytic area was quantified using Image J software (NIH, Bethesda, MD, US).

### MMPs promoter luciferase activity assay

For the luciferase assay, the reporter constructs of rat MMP2 (GenBank: DQ915967.1) and rat MMP9 (GenBank: AF148065.1) promoter were cloned into the pGL3 basic vector (Promega). VSMCs were transiently transfected with a total of 100 ng each of the luciferase reporter constructs using Lipofectamine 3000 (Invitrogen). To ensure efficient transfection, all wells were also co-transfected with a Renilla luciferase vector (pRL-TK; Promega). After 24 h transfection, the cells were treated with either negative control (DMSO), SPM, WPM, PAHs, or oxy-PAHs for 12 h. Luciferase activities were determined using the Dual-Luciferase Reporter Assay System (Promega) as described in the manufacturer’s protocol. Data were normalized by internal Renilla luciferase activity.

### Quantitative real-time PCR (qRT-PCR)

The expression levels of the various genes were analyzed by qRT-PCR. Cells were seeded into a 6well plate with glass coverslips at a density of 5 × 10^5^ cells/ml and cultured for 24 h. Cells were treated with negative control (0.1% DMSO), organic matters from PM, PAHs, or oxy-PAHs for 12 h. Total RNA was extracted using TRIzol lysis reagent (QIAGEN) according to the instructions provided by the manufacturer. The RNA concentration of each sample was measured by a spectrophotometer (Eppendorf) at 260 nm. Total RNA was subjected to reverse transcription using HelixCript™ 1st-Strand cDNA Synthesis Kit (NanoHelix). Real-time quantitative PCR with realHelix™ qPCR kit (NanoHelix) was performed by the SYBR Green method using an Applied Rotor-Gene 3000™. Gene expression was normalized to GAPDH. The relative mRNA expression levels were quantified and analyzed using Rotor-Gene 6 software (Corbett-research) using ^△△^Ct methods. Table 1 was primer sequences for qPCR.

**Table 1:** The sequence of primers used for real-time quantitative PCR

Gene	Primer sequence
GAPDH	Sense: 5′-ACAGGGCAGTGGGATACAGGT-3′ Antisense: 5′-AAACAGCAAAGGGCAACAAAG-3′
MMP2	Sense: 5′-ACGAGGACTCCCCTCTGCAT-3′ Antisense: 5′-AGGCCTTGGGTCAGGTTTAGA-3′
MMP9	Sense: 5′-CAAACAGGGCAGAAGACACC-3′ Antisense: 5′-CTCTGAGGGTGCTCCACCT-3′
MMP13	Sense: 5′-TCTGCACCCTCAGCAGGTTG-3′ Antisense: 5′-CATGAGGTCTCGGGATGGATG-3′
Nrf2	Sense: 5′-CTCTCTGGAGACGGCCATGACT-3′ Antisense: 5′-CTGGGCTGGGGACAGTGGTAG-3′
HO-1	Sense: 5′-TTGTCTCTCTGGAATGGAAGG-3′ Antisense: 5′-GGCTCAGAACAGCCGCCTCTA-3′
NQO1	Sense: 5′-CATTCTGAAAGGCTGGTTTGA-3′ Antisense: 5′-CTAGCTTTGATCTGGTTGTCAG-3′

### Immunoblot analysis

VSMCs were seeded into a 6well plate at a density of 5 × 10^5^ cells/ml and cultured for 24 h. Cells were treated with negative control (DMSO), ambient PM, or PAHs in a dose dependent manner for 24 h. Cells were washed once in PBS and lysed in RIPA buffer containing PMSF and phosphatase inhibitor. Protein concentrations were determined using the Bradford protein Assay. Proteins were separated in a 6–10% sodium dodecyl sulfate–polyacrylamide gel and transferred to a polyvinylidene difluoride membrane (Bio-Rad Laboratories, Inc.). After membrane blocking with Tris-buffered saline-Tween 20 (TBS-T, 0.1% Tween 20) containing 5% skim milk for 1 h at room temperature, the membrane was incubated with primary antibody overnight at 4 °C. The primary antibodies were used at the following dilutions in blocking buffer: MMP2 (1:4000), MMP9 (1:1000) (Abcam, Cambridge, MA, US), FAK (1:1000), p-FAK(Y397) (1:500), p-FAK(Y925) (1:500), Src (1:4000), p-Src (1:2000), Akt (1:4000), p-Akt (1:2000), ERK (1:3000), p-ERK (1:5000) (Cell Signaling, Beverly, MA, US), MMP13 (Novus, Centennial, CO, US, 1:1000), β-actin (Sigma, MO, US, 1:5,000). The membrane was washed five times with TBS-T for 5 min and incubated for 1 h at room temperature with secondary antibodies. After extensive washing, bands were detected by enhanced chemiluminescence reagent (ECL, BIONOTE, Animal Genetics Inc.). Band intensities were quantified using the Image J quantification software.

### Immunofluorescence staining

VSMCs were seeded into a 24well plate at a density of 3 × 10^4^ cells/ml and cultured for 24 h. Cells were fixed with 4% paraformaldehyde for 15 min and permeabilized with 0.2% Triton X-100 for 10 min at RT. After washing, cells were blocked with 2% BSA in PBS for 1 h, removed from the blocking solution, and incubated overnight at 4 °C with rabbit anti-Nrf2 (1:50) (Cell Signaling, Beverly, MA, US), rabbit anti-AhR (1:100) (BioWord, MN, US), rabbit-anti-FAK (1:100) (Cell Signaling, Beverly, MA, US), mouse anti-paxillin (1:300) (Sigma, MO, US), mouse anti-integrin β1 (1:100) (Sigma, MO, US) and actin stains with DyLight™ 594 Phalloidin (1:20) (Cell Signaling, Beverly, MA, US). Then, the cells were gently washed under the cover slip three times with PBS and mounted in fluoroshield™ mounting medium with DAPI (Cell Signaling, Beverly, MA, US). Cells were visualized under a laser scanning confocal microscope (Fluoview FV1000 confocal system, Olympus). To ensure blinded data analysis, immunofluorescence staining were performed by one person, and all images and samples were recorded by another person.

### Statistical analysis

All quantified data from at least triplicate samples were analyzed with GraphPad Prism 8.0 software. Data are expressed as mean ± SD. Statistical comparisons between the two groups were performed using Student's t-test. Statistical comparisons among multiple groups were performed using one-way ANOVA followed by Bonferroni post hoc test when the F statistic was significant. A two-tailed P < 0.05 was considered statistically significant.

## Data Availability

The datasets used and/or analyzed during the current study are available from the corresponding author on reasonable request.

## References

[1] Pang Y, Zhang B, Xing D, Shang J, Chen F, Kang H (2019). Increased risk of carotid atherosclerosis for long-term exposure to indoor coal-burning pollution in rural area, Hebei Province, China. Environ Pollut.

[2] Ranzani OT, Mila C, Sanchez M, Bhogadi S, Kulkarni B, Balakrishnan K (2020). Association between ambient and household air pollution with carotid intima-media thickness in peri-urban South India: CHAI-Project. Int J Epidemiol.

[3] Yang S, Lee SP, Park JB, Lee H, Kang SH, Lee SE (2019). PM2.5 concentration in the ambient air is a risk factor for the development of high-risk coronary plaques. Eur Heart J Cardiovasc Imaging.

[4] Jilani MH, Simon-Friedt B, Yahya T, Khan AY, Hassan SZ, Kash B (2020). Associations between particulate matter air pollution, presence and progression of subclinical coronary and carotid atherosclerosis: a systematic review. Atherosclerosis.

[5] Johnson MA, Steenland K, Piedrahita R, Clark ML, Pillarisetti A, Balakrishnan K (2020). Air pollutant exposure and stove use assessment methods for the household air pollution intervention network (HAPIN) trial. Environ Health Perspect.

[6] Wang S, Wang F, Yang L, Li Q, Huang Y, Cheng Z (2020). Effects of coal-fired PM2.5 on the expression levels of atherosclerosis-related proteins and the phosphorylation level of MAPK in ApoE(-/-) mice. BMC Pharmacol Toxicol.

[7] Miller MR, McLean SG, Duffin R, Lawal AO, Araujo JA, Shaw CA (2013). Diesel exhaust particulate increases the size and complexity of lesions in atherosclerotic mice. Part Fibre Toxicol.

[8] Song HS, Bang WG, Chung N, Cho YS, Kim YS, Cho MH (2003). Effect of chelators and reductants on the mobilization of metals from ambient particulate matter. Environ Sci Technol.

[9] Poursafa P, Moosazadeh M, Abedini E, Hajizadeh Y, Mansourian M, Pourzamani H (2017). A systematic review on the effects of polycyclic aromatic hydrocarbons on cardiometabolic impairment. Int J Prev Med.

[10] Brucker N, Charao MF, Moro AM, Ferrari P, Bubols G, Sauer E (2014). Atherosclerotic process in taxi drivers occupationally exposed to air pollution and co-morbidities. Environ Res.

[11] Niu X, Ho SSH, Ho KF, Huang Y, Sun J, Wang Q (2017). Atmospheric levels and cytotoxicity of polycyclic aromatic hydrocarbons and oxygenated-PAHs in PM2.5 in the Beijing-Tianjin-Hebei region. Environ Pollut.

[12] Simoneit BR, Medeiros PM, Didyk BM (2005). Combustion products of plastics as indicators for refuse burning in the atmosphere. Environ Sci Technol.

[13] Zielinska B, Sagebiel J, McDonald JD, Whitney K, Lawson DR (2004). Emission rates and comparative chemical composition from selected in-use diesel and gasoline-fueled vehicles. J Air Waste Manag Assoc.

[14] Wang L, Atkinson R, Arey J (2007). Formation of 9,10-phenanthrenequinone by atmospheric gas-phase reactions of phenanthrene. Atmos Environ.

[15] Barbas JT, Sigman ME, Dabestani R (1996). Photochemical oxidation of phenanthrene sorbed on silica gel. Environ Sci Technol.

[16] Stockfelt L, Andersson EM, Molnar P, Gidhagen L, Segersson D, Rosengren A (2017). Long-term effects of total and source-specific particulate air pollution on incident cardiovascular disease in Gothenburg, Sweden. Environ Res.

[17] Du Y, Xu X, Chu M, Guo Y, Wang J (2016). Air particulate matter and cardiovascular disease: the epidemiological, biomedical and clinical evidence. J Thorac Dis.

[18] Okona-Mensah KB, Battershill J, Boobis A, Fielder R (2005). An approach to investigating the importance of high potency polycyclic aromatic hydrocarbons (PAHs) in the induction of lung cancer by air pollution. Food Chem Toxicol.

[19] Pedersen DU, Durant JL, Penman BW, Crespi CL, Hemond HF, Lafleur AL (2004). Human-cell mutagens in respirable airborne particles in the northeastern United States. 1. Mutagenicity of fractionated samples. Environ Sci Technol.

[20] Ju S, Lim L, Jiao HY, Choi S, Jun JY, Ki YJ (2020). Oxygenated polycyclic aromatic hydrocarbons from ambient particulate matter induce electrophysiological instability in cardiomyocytes. Part Fibre Toxicol.

[21] Mill C, George SJ (2012). Wnt signalling in smooth muscle cells and its role in cardiovascular disorders. Cardiovasc Res.

[22] Shukla H, Lee HY, Koucheki A, Bibi HA, Gaje G, Sun X (2020). Targeting glutathione with the triterpenoid CDDO-Im protects against benzo-a-pyrene-1,6-quinone-induced cytotoxicity in endothelial cells. Mol Cell Biochem.

[23] Meng D, Lv DD, Zhuang X, Sun H, Fan L, Shi XL (2009). Benzo[a]pyrene induces expression of matrix metalloproteinases and cell migration and invasion of vascular smooth muscle cells. Toxicol Lett.

[24] Wang Z, Castresana MR, Newman WH (2004). Reactive oxygen species-sensitive p38 MAPK controls thrombin-induced migration of vascular smooth muscle cells. J Mol Cell Cardiol.

[25] Meng D, Lv DD, Fang J (2008). Insulin-like growth factor-I induces reactive oxygen species production and cell migration through Nox4 and Rac1 in vascular smooth muscle cells. Cardiovasc Res.

[26] Cabral-Pacheco GA, Garza-Veloz I, Castruita-De la Rosa C, Ramirez-Acuna JM, Perez-Romero BA, Guerrero-Rodriguez JF (2020). The roles of matrix metalloproteinases and their inhibitors in human diseases. Int J Mol Sci.

[27] Lin YC, Chen LH, Varadharajan T, Tsai MJ, Chia YC, Yuan TC (2014). Resveratrol inhibits glucose-induced migration of vascular smooth muscle cells mediated by focal adhesion kinase. Mol Nutr Food Res.

[28] Park HS, Quan KT, Han JH, Jung SH, Lee DH, Jo E (2017). Rubiarbonone C inhibits platelet-derived growth factor-induced proliferation and migration of vascular smooth muscle cells through the focal adhesion kinase, MAPK and STAT3 Tyr(705) signalling pathways. Br J Pharmacol.

[29] Zacharopoulou N, Kallergi G, Alkahtani S, Tsapara A, Alarifi S, Schmid E (2020). The histone demethylase KDM2B activates FAK and PI3K that control tumor cell motility. Cancer Biol Ther.

[30] Kawajiri K, Fujii-Kuriyama Y (2017). The aryl hydrocarbon receptor: a multifunctional chemical sensor for host defense and homeostatic maintenance. Exp Anim.

[31] Jones S (2004). An overview of the basic helix-loop-helix proteins. Genome Biol.

[32] Klein SG, Cambier S, Hennen J, Legay S, Serchi T, Nelissen I (2017). Endothelial responses of the alveolar barrier in vitro in a dose-controlled exposure to diesel exhaust particulate matter. Part Fibre Toxicol.

[33] Gao M, Ma Y, Luo J, Li D, Jiang M, Jiang Q (2021). The role of Nrf2 in the PM-induced vascular injury under real ambient particulate matter exposure in C57/B6 mice. Front Pharmacol.

[34] Levonen AL, Inkala M, Heikura T, Jauhiainen S, Jyrkkanen HK, Kansanen E (2007). Nrf2 gene transfer induces antioxidant enzymes and suppresses smooth muscle cell growth in vitro and reduces oxidative stress in rabbit aorta in vivo. Arterioscler Thromb Vasc Biol.

[35] Tomkiewicz C, Herry L, Bui LC, Metayer C, Bourdeloux M, Barouki R (2013). The aryl hydrocarbon receptor regulates focal adhesion sites through a non-genomic FAK/Src pathway. Oncogene.

[36] Vicente-Manzanares M, Horwitz AR (2011). Cell migration: an overview. Methods Mol Biol.

[37] Souza HP, Souza LC, Anastacio VM, Pereira AC, Junqueira ML, Krieger JE (2000). Vascular oxidant stress early after balloon injury: evidence for increased NAD(P)H oxidoreductase activity. Free Radic Biol Med.

[38] Dikalova A, Clempus R, Lassegue B, Cheng G, McCoy J, Dikalov S (2005). Nox1 overexpression potentiates angiotensin II-induced hypertension and vascular smooth muscle hypertrophy in transgenic mice. Circulation.

[39] Markovics A, Biró A, Kun-Nemes A, Fazekas M, Rácz AA, Paholcsek M (2020). Effect of anthocyanin-rich extract of sour cherry for hyperglycemia-induced inflammatory response and impaired endothelium-dependent vasodilation. Nutrients.

[40] San Martin A, Griendling KK (2010). Redox control of vascular smooth muscle migration. Antioxid Redox Signal.

[41] Hu T, Luan R, Zhang H, Lau WB, Wang Q, Zhang Y (2009). Hydrogen peroxide enhances osteopontin expression and matrix metalloproteinase activity in aortic vascular smooth muscle cells. Clin Exp Pharmacol Physiol.

[42] Jernigan NL, Walker BR, Resta TC (2008). Reactive oxygen species mediate RhoA/Rho kinase-induced Ca2+ sensitization in pulmonary vascular smooth muscle following chronic hypoxia. Am J Physiol Lung Cell Mol Physiol.

[43] Qi Y, Liang X, Dai F, Guan H, Sun J, Yao W (2020). RhoA/ROCK pathway activation is regulated by AT1 receptor and participates in smooth muscle migration and dedifferentiation via promoting actin cytoskeleton polymerization. Int J Mol Sci.

[44] Moon SK, Kang SK, Kim CH (2006). Reactive oxygen species mediates disialoganglioside GD3-induced inhibition of ERK1/2 and matrix metalloproteinase-9 expression in vascular smooth muscle cells. FASEB J.

[45] Yang SW, Lim L, Ju S, Choi DH, Song H (2015). Effects of matrix metalloproteinase 13 on vascular smooth muscle cells migration via Akt-ERK dependent pathway. Tissue Cell.

[46] Cirrincione AM, Pellegrini AD, Dominy JR, Benjamin ME, Utkina-Sosunova I, Lotti F (2020). Paclitaxel-induced peripheral neuropathy is caused by epidermal ROS and mitochondrial damage through conserved MMP-13 activation. Sci Rep.

[47] Al-Dhfyan A, Alhoshani A, Korashy HM (2017). Aryl hydrocarbon receptor/cytochrome P450 1A1 pathway mediates breast cancer stem cells expansion through PTEN inhibition and beta-Catenin and Akt activation. Mol Cancer.

[48] Parsons JT, Horwitz AR, Schwartz MA (2010). Cell adhesion: integrating cytoskeletal dynamics and cellular tension. Nat Rev Mol Cell Biol.

[49] Huttenlocher A, Horwitz AR (2011). Integrins in cell migration. Cold Spring Harb Perspect Biol.

[50] Cai WJ, Li MB, Wu X, Wu S, Zhu W, Chen D (2009). Activation of the integrins alpha 5beta 1 and alpha v beta 3 and focal adhesion kinase (FAK) during arteriogenesis. Mol Cell Biochem.

[51] Weise-Cross L, Sands MA, Sheak JR, Broughton BRS, Snow JB, Gonzalez Bosc LV (2018). Actin polymerization contributes to enhanced pulmonary vasoconstrictor reactivity after chronic hypoxia. Am J Physiol Heart Circ Physiol.

[52] MacKay CE, Knock GA (2015). Control of vascular smooth muscle function by Src-family kinases and reactive oxygen species in health and disease. J Physiol.

[53] Gray DL, Wallace LA, Brinkman MC, Buehler SS, La Londe C (2015). Respiratory and cardiovascular effects of metals in ambient particulate matter: a critical review. Rev Environ Contam Toxicol.

[54] Lee HH, Choi NR, Lim HB, Yi SM, Kim YP, Lee JY (2018). Characteristics of oxygenated PAHs in PM10 at Seoul, Korea. Atmos Pollut Res.

[55] Lim L, Yun JJ, Jeong JE, Wi AJ, Song H (2015). Inhibitory effects of nano-extract from *Dendropanax morbifera* on proliferation and migration of vascular smooth muscle cells. J Nanosci Nanotechnol.

